# Parental presence improves pediatric MRI success without sedation: a prospective randomized study

**DOI:** 10.3389/fped.2025.1559935

**Published:** 2025-06-23

**Authors:** Hideyuki Iwayama, Noriko Hayata, Mizuki Takagi, Ryohei Fukatsu, Kohei Kawahara, Hiroaki Somiya, Jun Sada, Shingo Numoto, Kiyoshi Yamakawa, Ryosuke Miyamoto, Hiromitsu Mori, Taichiro Muto, Hirokazu Kurahashi, Mio Ando, Makoto Endo, Wataru Ohashi, Sachiko Kitagawa, Yoshinori Ito, Akihisa Okumura

**Affiliations:** ^1^ Department of Pediatrics, Aichi Medical University School of Medicine, Nagakute, Aichi, Japan; ^2^ Department of Radiology, Aichi Medical University School of Medicine, Nagakute, Aichi, Japan; ^3^ Division of Biostatistics, Clinical Research Center, Aichi Medical University School of Medicine, Nagakute, Aichi, Japan; ^4^ Department of Pediatrics, Daiyukai General Hospital, Ichinomiya, Aichi, Japan

**Keywords:** non-sedated MRI, parental presence, pediatric imaging, success rate, randomized controlled study, image quality assessment

## Abstract

**Introduction:**

Magnetic resonance imaging (MRI) requires children to remain still for extended periods, often necessitating sedation, which carries risks and raises costs. Non-pharmacologic strategies such as video goggles, evening scheduling, mock MRI training, and child life specialist-led preparation have been explored. The effectiveness of parental presence, especially in younger children, remains underexamined.

**Methods:**

This prospective, single-center, randomized controlled trial included children aged 3–10 years referred for short stature evaluation. All were admitted for GH testing and underwent pituitary MRI if peak GH was ≤6.0 ng/ml. Participants were stratified by age (3–6 and 7–10 years) and randomized to parent present or absent groups using block randomization. MRI success was assessed in three steps: Step 1, completion of all sequences; Step 2, image quality (no, mild, or severe artifacts) evaluated blindly by two pediatricians; Step 3, final success defined as completion with no or mild artifacts. Exploratory variables included sibling number and crying during routine vaccinations.

**Results:**

Eighty children were enrolled, with 40 assigned to each group. Step 1: Completion rates were 30/40 (75.0%) in the parent present group and 22/40 (55.0%) in the parent absent group (*P* = 0.25). In children aged 3–6 years, completion was significantly higher in the parent present group (13/22, 59.1%) than in the parent absent group (4/22, 18.2%) (*P* = 0.012). Step 2: Among 52 who completed MRI, image quality was no/mild/severe artifact in 11/17/2 children (parent present) and 12/10/0 (parent absent) (*P* = 0.38). Step 3: Final success was achieved in 28/40 (70.0%) in the parent present group and 22/40 (55.0%) in the parent absent group (*P* = 0.25). In the 3–6-year subgroup, success was significantly higher in the parent present group (*P* = 0.012; OR = 6.50, 95% CI: 1.64–25.76). No difference was observed in the 7–10-year subgroup. Crying during vaccinations and sibling number were not associated with MRI success.

**Discussion:**

Parental presence significantly improved non-sedated MRI success in children aged 3–6 years. Compared to other interventions, it is simple, safe, low-cost, and requires no specialized resources, supporting its use as a first-line strategy in younger children.

## 1 Introduction

Magnetic resonance imaging (MRI) requires patients to remain still on the examination table for an extended period, which can be especially challenging for children. Sedation is commonly used to facilitate MRI in pediatric patients, but it carries potential risks and increases healthcare costs (1). Many parents and clinicians prefer to avoid sedation whenever possible to minimize post-procedural side effects (2).

Various non-pharmacologic strategies have been explored to reduce the need for sedation during MRI, depending on the child's age and developmental stage. The importance of considering children's cognitive and developmental readiness when communicating health-related information has been emphasized in theoretical frameworks by Piaget (3). For infants under one year of age, the feed-and-swaddle technique has proven effective (4). Older children, particularly those over six years, are often capable of following instructions and remaining still during imaging (5). However, this generally applies to children with typical development and may not hold true for those with developmental delays or disabilities. Preparation methods that have demonstrated benefit in selected populations include the use of video goggles (6, 7), scheduling MRI sessions after 7 p.m. (6), mock MRI training (8–10), and child life specialist (CLS) (7, 11). However, achieving high-quality, non-sedated MRI in children aged 3–6 years remains particularly difficult (7).

Parental presence has been studied in various pediatric settings, most notably during anesthesia induction (12–14) and painful procedures such as venipuncture (15, 16), with the aim of reducing anxiety in both parents and children and improving procedural cooperation. Guidelines from the Radiological Society of North America and MRI manufacturers recommend allowing parental presence in the scan room when feasible (17, 18). However, despite this recommendation, we found no prior studies that quantitatively evaluated the effectiveness of parental presence in MRI settings. In particular, evidence regarding its benefit in younger children remains lacking.

Identifying which children are likely to succeed with MRI without sedation can help optimize scheduling, reduce reliance on pharmacologic intervention, and improve the overall efficiency of pediatric imaging services. Several studies have reported that non-sedated MRI success can be predicted based on the child's developmental maturity or evaluations conducted by a parent or CLS (19, 20). However, these evaluation methods often require additional personnel and time, and a low-cost, practical approach to screening children for non-sedated MRI success has yet to be established.

This study aimed to evaluate the impact of parental presence on the success of MRI without sedation in children aged 3–10 years. We hypothesized that parental presence would improve the success rate, particularly in younger children who are less likely to tolerate the procedure on their own. Additionally, we considered easily obtainable factors such as the number of siblings and whether the child typically cries during routine vaccinations as potential indicators of baseline temperament or social adaptability that could be assessed quickly in clinical settings.

## 2 Materials and methods

### 2.1 Study design and participants

This prospective, single-center, randomized controlled trial was conducted between November 2018 and July 2022 at Aichi Medical University Hospital. Children were eligible if they met all of the following criteria: (a) referred for evaluation of short stature, (b) pituitary MRI required due to suspected growth hormone (GH) deficiency, and (c) aged 3–10 years.

Children were excluded if they had a diagnosed intellectual disability or neurodevelopmental disorder, or a congenital condition known to be associated with cognitive impairment, such as Down syndrome. Those with congenital anomalies unrelated to cognitive or behavioral development (e.g., ventricular septal defect) were not excluded. Eligibility was determined based on medical history available at the time of MRI scheduling. Children without a previously identified diagnosis were included even if developmental issues were later identified. The study was approved by the ethics committee of Aichi Medical University (approval number: 2018-H316), and written informed consent was obtained from all parents or legal guardians.

### 2.2 Clinical workflow and MRI preparation

All children were hospitalized for GH evaluation. On the afternoon of admission (Day 1), a left-hand radiograph was obtained to assess bone age. On the following morning (Day 2), GH secretion testing using clonidine was performed. For children aged 3–6 years, testing began at 6:30 a.m.; for those aged 7–10 years, it began at 9:00 a.m. If the peak GH concentration was ≤6.0 ng/ml, a pituitary MRI was scheduled for the afternoon of Day 2.

After the decision to proceed with MRI and prior to randomization, parents were interviewed to obtain information on the number of siblings and whether the child cried during routine vaccinations.

MRI preparation was conducted by a CLS when available, or otherwise by a pediatrician. Preparation included verbal reassurance, printed materials describing the MRI process, and photographs of the scanner. Children were shown a pamphlet explaining what to expect (e.g., loud noises), and were reassured that the procedure would not be painful. A soft toy and a wooden mock MRI scanner were also used to simulate the experience.

### 2.3 Randomization and parent instructions during procedures

Participants were stratified into two age groups (3–6 and 7–10 years) and randomly assigned to either the parent present or parent absent group using block randomization. Within each age group, blocks of four were created. The first participant in each block was assigned using a computer-generated random number (odd = parent absent; even = parent present); the second was assigned to the opposite group, the third to the same as the second, and the fourth to the same as the first, ensuring balanced allocation.

For children assigned to the parent present group, parents received a brief additional explanation before the scan. They were instructed to remain calm, speak gently to their child, and help prevent unnecessary movement. Parental contraindications (e.g., metal implants, pregnancy) were reviewed before MRI entry.

### 2.4 MRI room procedures and operational decision

All participants entered the MRI suite accompanied by a parent up to the entrance of the scan room. Children in the parent present group were accompanied into the scan room by a parent; those in the parent absent group were accompanied by a radiologic technologist. In the parent present group, the accompanying parent remained within reach of the child throughout the scan. A wooden chair was placed next to the MRI scanner, where the parent sat during the procedure. No distraction tools, such as video goggles or tablets, were used in either group.

If a child was unable to cooperate during positioning (e.g., by refusing to lie down, crying, or physically resisting), MRI was not initiated. In addition, if imaging could not begin within five minutes due to behavioral distress, or if excessive motion during scanning prevented image acquisition, the radiologic technologist was authorized to terminate the session. These cases were considered as failures to complete imaging. Children who failed to complete MRI were rescheduled for a sedated MRI on a later date as part of routine clinical care; however, these sedated MRIs were not included in the study analysis.

### 2.5 Pituitary MRI protocol

Pituitary MRI protocols were standardized across all participants, and sequence parameters are summarized in Supplementary Table S1. Imaging included sagittal, coronal, and axial T1-weighted sequences targeting the hypothalamic–pituitary region. The total imaging time was approximately 12 min, including six diagnostic sequences; an additional 1–2 min were needed for scan adjustments.

Parallel imaging techniques were used for all sequences, allowing approximately 40% scan time reduction. To reduce anxiety, quiet imaging techniques (Quiet Suite; Siemens Healthineers) were applied. The localizer scan, which is not diagnostic, was performed with maximal noise reduction. Subsequent sequences were optimized to maintain diagnostic quality while minimizing acoustic noise.

Three-dimensional T1-weighted imaging (e.g., MPRAGE) was not used due to its long acquisition time and sensitivity to motion, making it unsuitable for young children. AI-based image reconstruction, compressed sensing, and motion correction techniques were not used at the time of the study.

Scan planning was based on axial images visualizing the pituitary stalk. Sagittal images were acquired along the midline, and coronal images perpendicular to the stalk. The anatomical target was defined as the pituitary stalk and surrounding structures, including the hypothalamus and posterior pituitary. This target definition was independent of motion artifacts and not used to define MRI success.

Completion of imaging was defined as successful acquisition of all planned sequences, including those required to visualize the anatomical target. Scans interrupted before this point were considered failures to complete MRI.

### 2.6 MRI success criteria and image quality assessment

**Figure 1:**
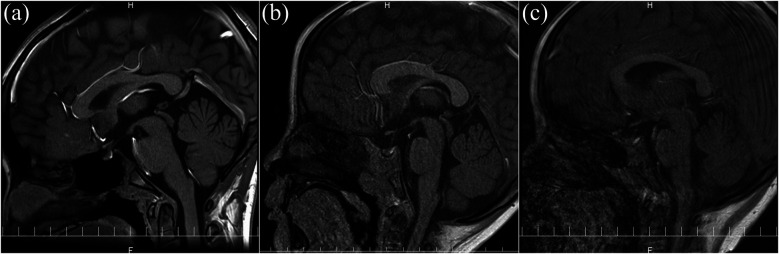
Representative images of the MRI quality: **(a)** no artifacts and fully evaluable; **(b)** mild artifacts and evaluable; **(c)** severe artifacts and not evaluable.

MRI success was evaluated in three steps:

**Step 1: Completion of Imaging**
Defined as acquisition of all planned sequences. Interrupted scans were classified as failures. Specific reasons for failure (e.g., refusal to enter, excessive movement) were not systematically recorded.
**Step 2: Image Quality Assessment**
Images were independently evaluated by two blinded pediatricians. Discrepancies were resolved by consensus. Image quality was categorized as: (a) no artifacts, (b) mild artifacts, or (c) severe artifacts (not evaluable). Figure 1 shows representative examples.
**Step 3: Final MRI Success Definition**
Defined as successful completion (Step 1) and image quality rated as no or mild artifacts (Step 2). Incomplete scans or those with severe artifacts were classified as failures. A summary of the success criteria is shown in Table 1.

**Table 1:** Definition of final MRI success and failure based on imaging completion (step 1) and image quality (step 2).

Step 1: Imaging completion	Step 2: Image quality		
	No artifact	Mild artifact	Severe artifact
Completed	Success	Success	Failure
Not completed	Failure	Failure	Failure

### 2.7 Statistics

Demographic and clinical characteristics—including age, sex, number of siblings, MRI preparation provider (CLS or pediatrician), and whether the child cried during routine vaccinations—were analyzed as potential predictors of MRI success.

Group comparisons were conducted using the Mann–Whitney *U* test for continuous variables and Fisher's exact test for categorical variables. Analyses were performed using EZR (Saitama Medical Center, Jichi Medical University, Saitama, Japan) (21), a graphical interface for R (The R Foundation for Statistical Computing, Vienna, Austria). A two-sided *P* value < 0.05 was considered statistically significant.

## 3 Results

### 3.1 Participant characteristics

The characteristics of the participants are shown in Table 2. A total of 80 children participated in the study: 40 were assigned to the parent present group [median age: 5.95 years, interquartile range (IQR): 4.55–8.14], and 40 to the parent absent group (median age: 6.66 years, IQR: 4.69–8.93). No significant differences were observed between the groups regarding the number of siblings or crying during routine vaccinations across all ages, or the provider of MRI preparation in the 3–6-year subgroup. Among children aged 7–10 years, the proportion of MRI preparation provided by a CLS vs. a pediatrician differed significantly between the parent present and parent absent groups (*P* = 0.04). However, all providers used the same explanatory materials and preparation tools, including printed pamphlets, photographs of the MRI scanner, and a wooden mock MRI. Therefore, we believe that this difference in personnel was unlikely to have influenced the imaging outcomes.

**Table 2:** The characteristics of the participants.

Age group		All participants			Ages 3–6 years			Ages 7–10 years		
Parental presence		Present (*n* = 40)	Absent (*n* = 40)	*P* value	Present (*n* = 22)	Absent (*n* = 22)	*P* value	Present (*n* = 18)	Absent (*n* = 18)	*P* value
Age (year) [mean, (IQR)]		5.95 (4.55–8.14)	6.66 (4.69–8.93)	0.41	4.64 (3.68–5.24)	5.01 (4.29–5.90)	0.22	8.39 (7.76–9.49)	9.10 (8.47–9.33)	0.29
Sex (M: F)		19:21	17:23	0.82	11:11	10:12	>0.99	8:10	7:11	>0.99
Number of siblings		1 (1–1)	1 (1–2)	0.60	1 (1–1)	1 (1–2)	0.51	1 (1–1)	1 (0.25–2)	0.99
Response to daily vaccinations	Crying	15	17	0.82	11	13	0.76	4	4	>0.99
	Not crying	25	23		11	9		14	14	
Preparation	CLS	25	19	0.26	11	12	>0.99	14	7	0.04
	Pediatrician	15	21		11	10		4	11	

CLS, child life specialist; IQR, interquartile range.

### 3.2 Step 1: completion of imaging

Completion of imaging was defined as the successful acquisition of all planned sequences, including those required to visualize the anatomical target. Scans interrupted before this point were considered failures to complete MRI. Imaging was completed in 30 of 40 children (75.0%) in the parent present group and in 22 of 40 (55.0%) in the parent absent group (*P* *=* 0.25) (Table 3). The remaining children were classified as failures at this stage. Specific reasons for failure (e.g., refusal to enter the scanner, early termination, or motion-related interruption) were not systematically recorded.

**Table 3:** Imaging completion (step 1), image quality (step 2), and final MRI success and failure (step 3).

	Age group	All participants				Age 3–6 years				Age 7–10 years			
Step	Parental presence	Present (*n* = 40)	Absent (*n* = 40)	*P* value	OR	Present (*n* = 22)	Absent (*n* = 22)	*P* value	OR	Present (*n* = 18)	Absent (*n* = 18)	*P* value	OR
1	Completed (*n*)	30	22	0.25		13	4	0.012		17	18	0.23	
	Not completed (*n*)	10	18			9	18			1	0		
2	No artifact (*n*)	11	12	0.38		4	1	>0.99		7	11	0.28	
	Mild artifact (*n*)	17	10			9	3			8	7		
	Severe artifact (*n*)	2	0			0	0			2	0		
3	Success (*n*, %)	28 (70.0%)	22 (55.0%)	0.25	1.909 (0.761–4.788)	13 (59.1%)	4 (18.2%)	0.012	6.500 (1.640–25.760)	15 (83.3%)	18 (100%)	0.23	–
	Failure (*n*, %)	12 (30.0%)	18 (45.0%)			9 (40.9%)	18 (81.8%)			3 (16.7%)	0 (0%)		

OR, Odds ratios are calculated for success in the parent absent group relative to the parent present group. Odds ratio was not calculable for the 7–10-year subgroup due to a zero-cell count.

In the 3–6-year subgroup, imaging was completed in 13 of 22 children (59.1%) in the parent present group and in 4 of 22 (18.2%) in the parent absent group (*P* *=* 0.012). In the 7–10-year subgroup, imaging was completed in 17 of 18 (94.4%) in the parent present group and in 18 of 18 (100%) in the parent absent group (*P* *=* 0.23).

### 3.3 Step 2: image quality among completed cases

Among the 52 children who completed imaging, image quality was evaluated and classified into three categories: no artifacts, mild artifacts, and severe artifacts. In the parent present group (*n* = 30), 11 children had no artifacts, 17 had mild artifacts, and 2 had severe artifacts. In the parent absent group (*n* = 22), 12 children had no artifacts, 10 had mild artifacts, and none had severe artifacts (*P* *=* 0.38 for comparison of severe artifacts between groups).

In the 3–6-year subgroup, no severe artifacts were observed in either group (*P* *>* 0.99). In the 7–10-year subgroup, severe artifacts were observed in 2 of 17 children in the parent present group and in none of the 18 children in the parent absent group (*P* *=* 0.28).

### 3.4 Step 3: final MRI success rate

Final success was defined as completion of imaging (Step 1) and acquisition of images with no or mild artifacts (Step 2) (Table 1). Overall, 28 of 40 children (70.0%) in the parent present group achieved final success, compared to 22 of 40 (55.0%) in the parent absent group (*P* *=* 0.25; odds ratio = 1.909, 95% confidence interval: 0.761–4.788).

In the 3–6-year subgroup, final success was achieved in 13 of 22 children (59.1%) in the parent present group and in 4 of 22 (18.2%) in the parent absent group (*P* *=* 0.012; odds ratio = 6.500, 95% confidence interval: 1.640–25.760).

In the 7–10-year subgroup, final success was achieved in 15 of 18 (83.3%) in the parent present group and in 18 of 18 (100%) in the parent absent group (*P* *=* 0.23; odds ratio not calculable due to zero failure in the parent absent group).

## 4 Discussion

This study demonstrated that parental presence significantly improved the success rate of non-sedated MRI in children aged 3–6 years but not in those aged 7–10 years. This effect was observed in the final success rate (Step 3), which was defined as both the completion of MRI and acquisition of evaluable images without severe artifacts. In the younger age group, the final success rate in the parent present group (59.1%) was significantly higher than that in the parent absent group (18.2%), with an odds ratio of 6.500 (95% CI: 1.640–25.760). However, no significant difference was found in children aged 7–10 years, among whom the success rate was high regardless of parental presence.

### 4.1 Age-dependent effectiveness of parental presence

Previous studies have reported that increasing age is significantly associated with the success of MRI without sedation (7). Consistently, our study found a higher success rate in children aged 7–10 years compared to those aged 3–6 years, regardless of parental presence. This indicates that patient age itself is a key determinant of procedural cooperation, likely reflecting cognitive maturity and behavioral regulation capacity.

The success of non-sedated MRI is closely related to the developmental stage of the child. According to Piaget's theory of cognitive development, children progress through four stages: the sensorimotor stage (birth to 2 years), pre-operational stage (2–7 years), concrete operational stage (7–11 years), and formal operational stage (11–16 years) (3). During the pre-operational stage (2–7 years), children's understanding is still egocentric and concrete, making it difficult for them to follow complex instructions or remain still for extended periods. In contrast, by the concrete operational stage (7–11 years), children become capable of understanding rules, sequencing events, and following instructions. These cognitive abilities enable them to better tolerate the demands of MRI procedures without sedation (5).

This age-dependent effect is consistent with prior studies using other non-pharmacologic interventions. One study reported that the use of video goggles significantly reduced the need for sedation in children aged 3–10 years, but not in younger children aged 0–2 years (*P* < 0.001 for 3–10 years vs. *P* = 0.81 for 0–2 years) (6). This indicates that non-pharmacologic interventions, including parental presence or audiovisual distraction, may have age-dependent effects on the success of non-sedated MRI.

Thus, when attempting MRI without sedation, the child's developmental stage should be carefully considered, as it does not always align with chronological age. While parental presence may benefit children with less mature behavioral regulation, age-appropriate communication and psychological preparation remain essential across developmental levels.

### 4.2 Comparison with parental presence in other procedures

Parental presence has been studied in various pediatric settings, most notably during anesthesia induction and painful procedures such as venipuncture. In the context of anesthesia induction, some studies have reported that parental presence can reduce parents' and children's anxiety (12), while others have found no significant effects on anxiety levels in either group (13, 14). This inconsistency may be partly explained by the nature of the procedure: during anesthesia induction, the child remains awake only briefly before losing consciousness, leaving a narrow window for behavioral intervention. Moreover, success typically does not depend on the child remaining still or calm for an extended period, which may limit the potential benefit of parental presence.

In painful procedures, parental presence has shown more consistent benefits. A systematic review concluded that it was associated with reduced self-reported pain and physiological stress responses in children (15). However, its effect on anxiety and behavioral distress was less clear. Similarly, a randomized trial found that parental presence significantly reduced pain scores during invasive procedures, but did not consistently improve anxiety levels (16). These findings suggest that while parental presence may help modulate pain perception, it may be less effective in addressing emotional distress when the procedure itself involves unavoidable pain.

In contrast, MRI scanning is painless but demands prolonged immobility in a loud, enclosed environment, often without physical contact with a caregiver. Importantly, unlike anesthesia induction or painful procedures, MRI provides a longer duration during which a parent can actively support the child through verbal reassurance and emotional presence. For younger children who lack fully developed self-regulation skills, this opportunity for continuous support may be particularly valuable.

Our findings support this interpretation. In our study, parental presence was associated with a significantly higher success rate for non-sedated MRI in children aged 3–6 years. This effect may have been amplified by the brief, structured instruction provided to parents before the scan, which encouraged calmness, gentle communication, and helping the child remain still. These results suggest that, in the context of pediatric MRI, parental presence should be regarded not as a passive allowance but as an active, age-appropriate support strategy.

### 4.3 Comparison with other non-pharmacologic interventions

Several non-pharmacologic strategies have been explored to improve the success of MRI without sedation in children, including the use of video goggles, scheduling MRI sessions after 7 p.m., mock MRI training, and child life preparation. While each of these methods has demonstrated some benefit, they also present practical limitations in terms of cost, availability, and staffing.

Audiovisual distraction systems, such as video goggles, have been shown to reduce the need for sedation, particularly in children aged 3–10 years (6). This approach, however, requires specialized equipment and may not be feasible in all clinical environments. Evening MRI scheduling (after 7 p.m.) has also improved non-sedated MRI success in some studies, possibly by reducing daytime distractions or aligning with children's natural sleepiness (6). Nevertheless, these strategies place additional burdens on staffing and institutional resources.

Mock MRI training has been shown to improve the success rate of non-sedated MRI in children. Several studies have reported that the use of simulator-based preparation, including commercial or low-cost mock scanners, can significantly reduce the need for sedation (8–10). However, such programs require dedicated equipment and personnel for explanation and practice, which may limit their feasibility in routine clinical settings.

A multidisciplinary approach, including CLS support and the use of age-appropriate tools such as video goggles, was reported to achieve an 82% success rate for nonsedated neuroradiologic MRI in children aged 1–7 years (7). Similarly, in another study, CLS-led preparation reduced general anesthesia use from 45.4%–35.4% in children aged 5–10 years (11). However, the use of supportive devices such as video goggles (7) and the administration of diazepam as conscious anxiolysis (11) make it difficult to isolate the independent effect of CLS support on non-sedated MRI success.

In contrast, parental presence is a low-cost, immediately implementable strategy that requires no specialized resources. It leverages the caregiver's natural relationship with the child and allows for continuous, real-time emotional support. Our findings suggest that parental presence may serve as a practical first-line approach to improve the success of non-sedated MRI in young children.

### 4.4 Predictive factors associated with non-sedated MRI

Identifying children who are likely to tolerate MRI without sedation is essential for reducing unnecessary pharmacologic intervention and improving clinical efficiency. Prior studies have demonstrated that behavioral and developmental characteristics—such as attention regulation, adaptability, and parental expectations—are stronger predictors of procedural success than chronological age alone (19, 20). These findings emphasize that developmental maturity is a key determinant of non-sedated MRI success.

In our study, we evaluated two easily obtainable variables—crying during routine vaccinations and the number of siblings—as simple proxies for temperament and social adaptability. However, neither showed a significant association with MRI success. Compared with more comprehensive behavioral assessments used in previous research, these proxy measures may lack the sensitivity and specificity required to predict cooperation. This contrast highlights the need for more robust and practical screening tools to assess developmental readiness for non-sedated MRI.

### 4.5 Limitations in this study

This study has several limitations. First, participants represented a relatively homogeneous population: children admitted for evaluation of short stature who underwent pituitary MRI as part of a standardized endocrine workup. Thus, the findings may not be generalizable to other pediatric populations, such as those with pain, acute illness, neurodevelopmental conditions, or those requiring prolonged MRI protocols. A multicenter study including more diverse populations is needed to assess broader applicability.

Second, the image quality grading method, although practical, was not formally validated. Our study used a three-tier scale similar to that used in infant MRI studies (4), while other studies have used different grading systems (8–11), limiting comparability. Developing a standardized, validated scoring method will improve reproducibility in future studies.

Finally, although we examined possible behavioral predictors of MRI success, the findings and their implications have been discussed in detail in Section 4.4.

## 5 Conclusion

Parental presence significantly improved the success rate of MRI without sedation in children aged 3–6 years, whereas no additional benefit was observed in older children. Owing to its simplicity, safety, and low cost, parental presence constitutes an effective and feasible first-line strategy to enhance the success of non-sedated MRI in young children, prior to implementing more resource-intensive interventions.

## Data Availability

The raw data supporting the conclusions of this article will be made available by the authors, without undue reservation.
