# Supplementary material for: Parental presence improves pediatric MRI success without sedation: a prospective randomized study
Source: Front Pediatr. 2025 Jun 23;13:1559935. doi: 10.3389/fped.2025.1559935 (PMC12230062; doi:10.3389/fped.2025.1559935)
Supplement: Supplementary file 1 [file Table1.docx]

Supplementary Table S1. Sequence parameters for pituitary MRI used in this study

| 1.5T MRI Scanner (Magnetom Avanto fit, Magnetom Aera; Siemens) | | | | |  |  |  |  |  |  |
| --- | --- | --- | --- | --- | --- | --- | --- | --- | --- | --- |
| Sequence | Orientation | Focus | Sequence Type | TR/TE  (ms) | Slices  (n) | Thickness  (mm) | FA  (degree) | FOV  (mm) | Matrix  (pixels) | Scan Time  (min:s) |
| localizer | 3 planes | whole brain | 2D-Turbo Spin Echo | 600/18 | 9 | 8 | 150 | 300x215 | 192x97 | 0:25 |
| T2WI_tra | transverse | whole brain | 2D-Turbo Spin Echo | 6000/120 | 30 | 4 | 150 | 200x181 | 320x203 | 1:50 |
| T1WI_tra | transverse | pituitary | 2D-Gradient Eho | 300/4.76 | 13 | 2.5 | 70 | 160x160 | 256x243 | 1:45 |
| T1WI_sag | sagittal | pituitary | 2D-Gradient Eho | 300/4.76 | 13 | 2.5 | 70 | 160x161 | 256x243 | 1:45 |
| T2WI_sag | sagittal | pituitary | 2D-Turbo Spin Echo | 4500/100 | 13 | 2.5 | 150 | 160x162 | 256x205 | 1:54 |
| T1WI_cor | coronal | pituitary | 2D-Gradient Eho | 300/4.76 | 13 | 2.5 | 70 | 160x163 | 256x243 | 1:45 |
| T2WI_cor | coronal | pituitary | 2D-Turbo Spin Echo | 4500/100 | 13 | 2.5 | 150 | 160x162 | 256x205 | 1:54 |
|  |  |  |  |  |  |  |  |  |  |  |
| 3T MRI Scanner (Magnetom Skyra) | | | |  |  |  |  |  |  |  |
| Sequence | Orientation | Focus | Sequence Type | TR/TE  (ms) | Slices  (n) | Thickness  (mm) | FA  (degree) | FOV  (mm) | Matrix  (pixels) | Scan Time  (min:s) |
| localizer | 3 planes | whole brain | 2D-Turbo Spin Echo | 600/15 | 9 | 5 | 140 | 240x195 | 192x117 | 0:25 |
| T2WI_tra | transverse | whole brain | 2D-Turbo Spin Echo | 6500/115 | 30 | 4 | 150 | 220x182 | 352x232 | 1:34 |
| T1WI_tra | transverse | pituitary | 2D-Gradient Eho | 330/2.7 | 15 | 2.5 | 80 | 160x160 | 288x274 | 1:49 |
| T1WI_sag | sagittal | pituitary | 2D-Gradient Eho | 330/2.7 | 15 | 2.5 | 80 | 160x160 | 288x274 | 1:49 |
| T2WI_sag | sagittal | pituitary | 2D-Turbo Spin Echo | 5000/103 | 15 | 2.5 | 150 | 160x160 | 288x230 | 1:40 |
| T1WI_cor | coronal | pituitary | 2D-Gradient Eho | 330/2.7 | 15 | 2.5 | 80 | 160x160 | 288x274 | 1:55 |
| T2WI_cor | coronal | pituitary | 2D-Turbo Spin Echo | 5000/103 | 15 | 2.5 | 150 | 160x160 | 288x230 | 1:40 |

FA: flip angle; FOV: field of view; TE: echo time; TR: repetition time
